# Pax5 Negatively Regulates Osteoclastogenesis through Downregulation of Blimp1

**DOI:** 10.3390/ijms22042097

**Published:** 2021-02-20

**Authors:** Jiyeon Yu, Sumi Kim, Nari Lee, Hyoeun Jeon, Jun Lee, Masamichi Takami, Jaerang Rho

**Affiliations:** 1 Department of Microbiology and Molecular Biology, College of Bioscience and Biotechnology, Chungnam National University, Daejeon 34134, Korea; julmooni@hanmail.net (J.Y.); na12423@naver.com (S.K.); leenari6463@naver.com (N.L.); whyoeun@naver.com (H.J.); 2 Department of Oral and Maxillofacial Surgery, School of Dentistry, College of Dentistry, Wonkwang University, Iksan 54538, Korea; omslee@wku.ac.kr; 3 Department of Pharmacology, School of Dentistry, Showa University, 1-5-8 Hatanodai, Shinagawaku 142-8555, Japan; takami@dent.showa-u.ac.jp

**Keywords:** Pax5, BSAP, Blimp1, PRDM1, osteoclast, osteoclastogenesis

## Abstract

Paired box protein 5 (Pax5) is a crucial transcription factor responsible for B-cell lineage specification and commitment. In this study, we identified a negative role of Pax5 in osteoclastogenesis. The expression of Pax5 was time-dependently downregulated by receptor activator of nuclear factor kappa B (RANK) ligand (RANKL) stimulation in osteoclastogenesis. Osteoclast (OC) differentiation and bone resorption were inhibited (68.9% and 48% reductions, respectively) by forced expression of Pax5 in OC lineage cells. Pax5 led to the induction of antiosteoclastogenic factors through downregulation of B lymphocyte-induced maturation protein 1 (Blimp1). To examine the negative role of Pax5 in vivo, we generated Pax5 transgenic (Pax5^Tg^) mice expressing the human Pax5 transgene under the control of the tartrate-resistant acid phosphatase (TRAP) promoter, which is expressed mainly in OC lineage cells. OC differentiation and bone resorption were inhibited (54.2–76.9% and 24.0–26.2% reductions, respectively) in Pax5^Tg^ mice, thereby contributing to the osteopetrotic-like bone phenotype characterized by increased bone mineral density (13.0–13.6% higher), trabecular bone volume fraction (32.5–38.1% higher), trabecular thickness (8.4–9.0% higher), and trabecular number (25.5–26.7% higher) and decreased trabecular spacing (9.3–10.4% lower) compared to wild-type control mice. Furthermore, the number of OCs was decreased (48.8–65.3% reduction) in Pax5^Tg^ mice. These findings indicate that Pax5 plays a negative role in OC lineage specification and commitment through Blimp1 downregulation. Thus, our data suggest that the Pax5–Blimp1 axis is crucial for the regulation of RANKL-induced osteoclastogenesis.

## 1. Introduction

The strength and rigidity of bone are maintained by a proper homeostatic balance between bone-forming osteoblasts and bone-resorbing osteoclasts (OCs) [1,2]. As the only cells exhibiting bone resorption activity in vivo, OCs are clinically important; OCs lead to excess bone destruction in bone loss-associated disorders, such as rheumatoid arthritis, periodontitis, metastatic cancer, and osteoporosis [3,4].

OCs are derived from hematopoietic precursors in the bone marrow through the process of OC differentiation, also called osteoclastogenesis. Osteoclastogenesis is mediated mainly by the stimulation of receptor activator of nuclear factor kappa B (RANK) ligand (RANKL) and macrophage-colony stimulating factor (M-CSF). In particular, the crucial roles of RANKL, the primary cytokine involved in osteoclastogenesis, have been well elucidated [1,2,5,6]. Upon RANKL stimulation, the cytoplasmic tail of RANK recruits tumor necrosis factor receptor-associated factors, leading to the activation of downstream signaling pathways and thereby activating osteoclastogenic transcription factors, such as nuclear factor κB (NF-κB), activator protein-1 (AP-1), and nuclear factor of activated T cells c1 (NFATc1). NFATc1, the master transcription factor for osteoclastogenesis, is directly involved in the induction of osteoclastogenic markers, such as tartrate-resistant acid phosphatase (TRAP), dendritic cell-specific transmembrane protein (DC-STAMP), cathepsin K, Atp6V0d2, and OC-associated receptor (OSCAR). In addition, activation of NFATc1 can reduce the expression of antiosteoclastogenic factors, such as B-cell lymphoma 6 (Bcl6), interferon regulatory factor 8 (IRF8), and v-Maf musculoaponeurotic fibrosarcoma oncogene family homolog B (MafB), by inducing the B lymphocyte-induced maturation protein1 (Blimp1) in osteoclastogenesis [1,2,5,6].

Paired box protein 5 (Pax5), also called B-cell lineage-specific activator protein, is a crucial transcription factor responsible for cell fate determination during hematopoiesis [7]. Pax5 expression is maintained from the early B220^+^ pro-B-cell stage to the mature B-cell stage but not in the terminally differentiated plasma cell stage [8,9]. Pax5 represses the expression of Blimp1, a key transcriptional regulator of B-cell lineage commitment, thereby inhibiting the terminal differentiation of plasma cells [10,11,12]. Indeed, Pax5 deficiency promotes plasma cell differentiation [13]. Thus, Pax5 is a crucial factor in maintaining B-cell lineage identity [7]. Interestingly, it has been reported that pro-B cells derived from Pax5-deficient mice (Pax5^−/−^) can differentiate into not only OCs but also macrophages, dendritic cells, granulocytes, and natural killer cells in vitro, while Pax5^−/−^ pro-B cells cannot differentiate into mature B cells [14]. OC precursors derived from Pax5^−/−^ pro-B cells are highly enriched in the spleen in Pax5^−/−^ mice [14,15]. Furthermore, Pax5^−/−^ mice exhibit severe osteopenia with a loss of bone mass of more than 60% and an increase in the OC number [15]. Thus, the role of Pax5 may be closely linked to the regulation of osteoclastogenesis. However, the precise role of Pax5 in the regulation of osteoclastogenesis remains unclear at the molecular level.

In this study, we identified the role of Pax5 in the regulation of osteoclastogenesis. Osteoclastogenesis was reduced by forced expression of Pax5. Pax5 expression led to the induction of antiosteoclastogenic factors, such as Bcl6, IRF8, and MafB, through downregulation of Blimp1. Pax5 transgenic (Pax5^Tg^) mice expressing the human Pax5 transgene under the control of the *TRAP* promoter exhibited an osteopetrotic-like bone phenotype due to reduced OC formation. Thus, our data suggest that the regulation of the Pax5–Blimp1 axis is crucial for the regulation of RANKL-induced osteoclastogenesis.

## 2. Results

### 2.1. Pax5 Expression Is Downregulated during RANKL-Induced Osteoclastogenesis

**Figure 1:**
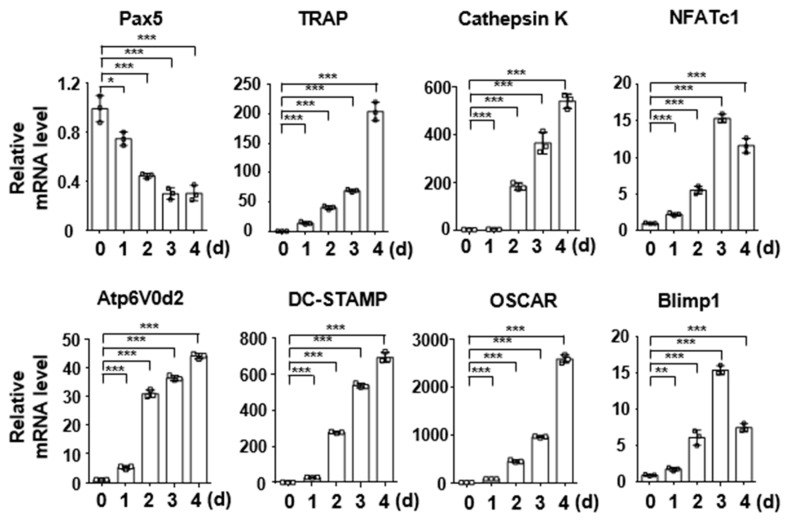
Paired box protein 5 (Pax5) is downregulated during osteoclastogenesis. Bone marrow-derived macrophages (BMMs) were differentiated with macrophage-colony stimulating factor (M-CSF) (50 ng/mL) and receptor activator of nuclear factor kappa B (RANK) ligand (RANKL) (100 ng/mL) for 4 days. Total RNA was isolated from cultured cells in triplicate and analyzed by real-time PCR using specific primers for Pax5, tartrate-resistant acid phosphatase (TRAP), cathepsin K, nuclear factor of activated T cells c1 (NFATc1), Atp6V0d2, dendritic cell-specific transmembrane protein (DC-STAMP), osteoclast (OC)-associated receptor (OSCAR), and B lymphocyte-induced maturation protein1 (Blimp1). β-Actin was used as the control. All points and error bars represent the mean ± SD of triplicate real-time PCRs. * *p* < 0.05, ** *p* < 0.01, *** *p* < 0.001.

Pax5^−/−^ mice exhibit severe osteopenia with an increase in the OC number and acceleration of bone loss [14,15]. Thus, we first examined whether Pax5 expression is downregulated during osteoclastogenesis. To evaluate Pax5 expression, bone marrow-derived macrophages (BMMs) were differentiated from bone marrow cells by culture with M-CSF (150 ng/mL) for 2 days and were then further differentiated into OCs by RANKL (100 ng/mL) stimulation in the presence of M-CSF (50 ng/mL) for 4 days. We examined whether Pax5 expression was abundantly maintained in BMMs on day 0 (Figure 1). During osteoclastogenesis, Pax5 expression was rapidly reduced by RANKL stimulation after day 1 (the early stage of OC differentiation) and almost returned to the basal level by day 3 (the later maturation stage), while the expression of OC markers, such as TRAP, cathepsin K, NFATc1, Atp6V0d2, DC-STAMP, OSCAR, and Blimp1, was enhanced in a RANKL-dependent manner (Figure 1). These results indicate that downregulation of Pax5 during osteoclastogenesis might be closely linked to the regulation of OC differentiation and OC marker expression.

### 2.2. Pax5 Negatively Regulates RANKL-Induced Osteoclastogenesis

**Figure 2:**
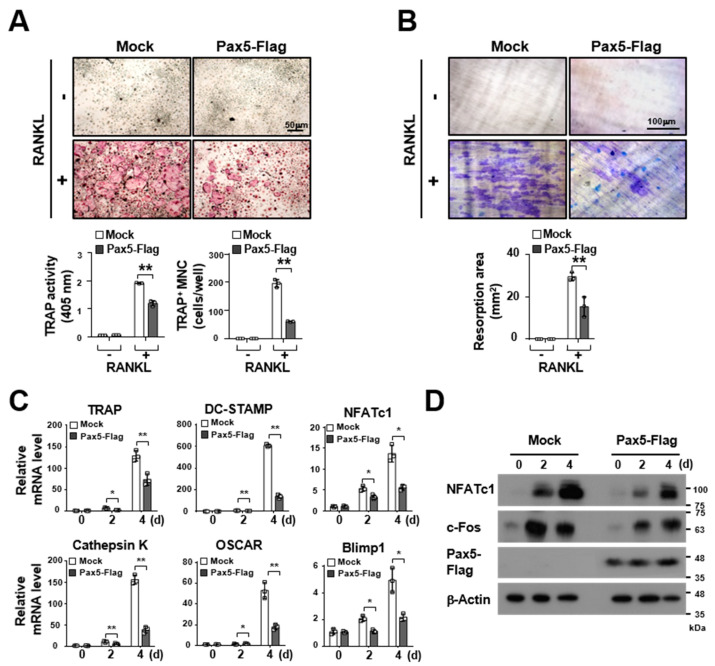
Osteoclastogenesis is downregulated by forced expression of Pax5. (**A**) Inhibitory effect of Pax5 overexpression on RANKL-induced osteoclastogenesis. BMMs expressing the Flag-tagged Pax5 transgene were generated by retroviral infection and puromycin selection, and puromycin-resistant BMMs expressing Flag-tagged Pax5 were differentiated into OCs by treatment with M-CSF (50 ng/mL) and RANKL (100 ng/mL) for 4 days. The mature OCs were photographed (top panel (scale bar, 50 μm)) after TRAP staining (original magnification, 50×). For the TRAP solution assay, the absorbance of OC samples was measured at 405 nm (bottom left panel). TRAP-positive multinucleated OCs (TRAP^+^ MNCs) with more than three nuclei were counted (bottom right panel). An empty control vector was used as the mock. (**B**) The inhibitory effect of Pax5 overexpression on resorption pit formation. Resorption pits on bone slices were visualized (top panel (scale bar, 100 μm)) by 1% toluidine blue staining (original magnification, 50×). The summary data from the resorption pit assays are shown in the bottom panel. (**C**) Downregulation of osteoclastogenic markers by Pax5 overexpression. Total RNA was isolated from BMM-derived OCs and analyzed by RT-PCR. The messenger RNA (mRNA) levels were normalized to those of β-actin. (**D**) Reduced protein levels of osteoclastogenic factors induced by Pax5 overexpression. The total cell lysates of OCs were analyzed by immunoblotting using antibodies against NFATc1 and c-Fos. β-Actin was used as the loading control. * *p* < 0.05, ** *p* < 0.01.

**Figure 3:**
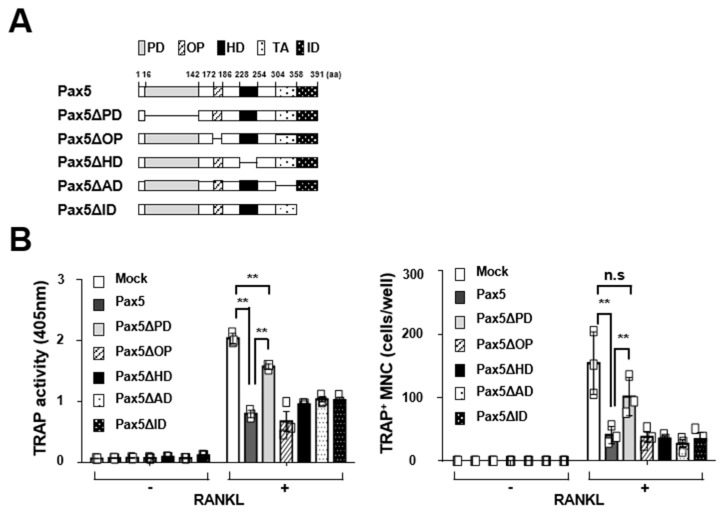
Inhibitory effect of Pax5 deletion mutants on osteoclastogenesis. (**A**) Schematic diagram of Pax5 deletion mutants. The domains of Pax5 are indicated: PD, paired domain; OP, octapeptide; HD, homeodomain; TA, transactivation domain; ID, inhibitory domain. Internal deletions are shown with lines. The amino-acid numbers are shown. (**B**) The effect of internal deletion mutants of Pax5 on osteoclastogenesis. BMMs expressing Flag-tagged Pax5 deletion mutants were selected with puromycin, and puromycin-resistant BMMs were differentiated into OCs with M-CSF (50 ng/mL) and RANKL (100 ng/mL) for 4 days. For the TRAP solution assay, the absorbance of OC samples was measured at 405 nm (bottom left panel). The TRAP^+^ MNCs with more than three nuclei were counted (bottom right panel). An empty control vector was used as the mock. ** *p* < 0.01, n.s. not significant.

We next determined whether the expression of Pax5 is involved in RANKL-induced osteoclastogenesis. To examine the role of Pax5 in osteoclastogenesis, Pax5 was overexpressed in BMMs using a retroviral gene transfer system, and Pax5-transduced BMMs were then differentiated into OCs by RANKL stimulation. TRAP activity in OCs was significantly inhibited (37.7% reduction on day 4, *p <* 0.01) by forced expression of Pax5 in the TRAP solution assay, and the number of TRAP^+^ multinucleated OCs (MNCs) was reduced (68.9% reduction on day 4, *p <* 0.01) in Pax5-overexpressing OCs (Figure 2A). Furthermore, the resorption area was decreased (48.0% reduction on day 4, *p <* 0.01) by Pax5 overexpression in OCs (Figure 2B). Consistent with these results, the messenger RNA (mRNA) and protein levels of OC markers were significantly decreased in Pax5-overexpressing OCs during osteoclastogenesis (Figure 2C,D). In analysis of deletion mutants, OC formation assays revealed that OC differentiation was partially restored upon loss of the conserved DNA-binding paired domain (PD) in Pax5, indicating that the DNA-binding activity of Pax5 is negatively involved in RANKL-induced osteoclastogenesis (Figure 3). Taken together, these results indicate that Pax5 expression is negatively involved in the regulation of RANKL-induced osteoclastogenesis.

### 2.3. Pax5 Enhances the Expression of Antiosteoclastogenic Factors through Downregulation of Blimp1

**Figure 4:**
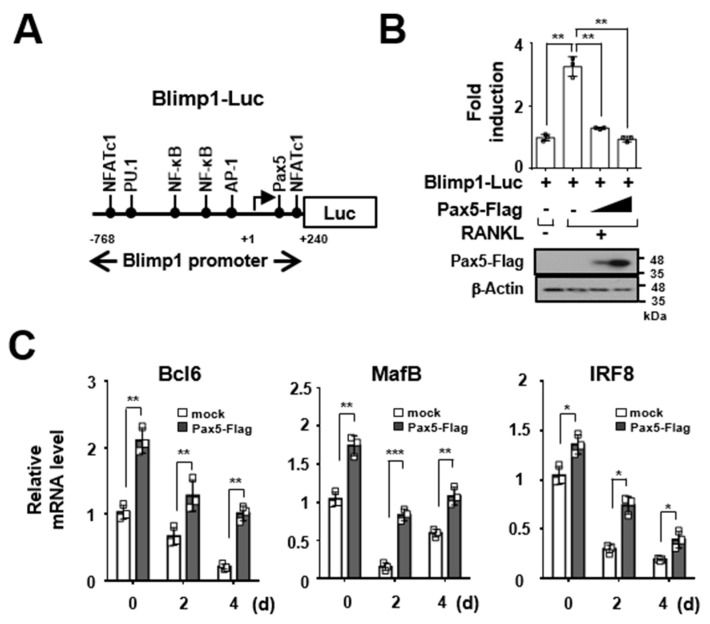
Pax5 induces the expression of antiosteoclastogenic factors through downregulation of Blimp1. (**A**) Schematic diagram of the luciferase reporter vector containing the Blimp1 promoter (Blimp1-Luc). The nucleotide positions are numbered according to the transcription start site, and the putative transcription factor binding sites are shown. (**B**) Inhibitory effect of Pax5 on RANKL-induced Blimp1-Luc reporter activity. RAW264.7 cells were cotransfected with the Flag-tagged Pax5 expression plasmid (Pax5-Flag (0.2–1.0 μg)), reporter plasmids (Blimp1-Luc (0.1 μg), and pcDNA3.1HisLacZ (0.13 μg)). The transfected cells were stimulated with RANKL (100 ng/mL) for 2 days and subjected to a luciferase reporter assay. The expression of Pax5 was analyzed by immunoblotting with anti-Flag antibodies. β-Actin was used as the loading control. (**C**) Effect of Pax5 on the expression of antiosteoclastogenic factors. BMMs expressing the Flag-tagged Pax5 transgene were differentiated into OCs in triplicate by treatment with M-CSF (50 ng/mL) and RANKL (100 ng/mL) for 4 days. The mature OCs were analyzed by RT-PCR. The mRNA levels were normalized to those of β-actin. An empty control vector was used as the mock. All points and error bars represent the mean ± SD of triplicate real-time PCRs. * *p* < 0.05, ** *p* < 0.01, *** *p* < 0.001.

Pax5 represses Blimp1 expression through direct binding to the Blimp1 promoter in human leukocytes [11]. Thus, we examined whether RANKL-induced Blimp1 promoter activity is negatively regulated by Pax5. To address this possibility, we constructed a luciferase reporter vector containing the murine Blimp1 promoter (Blimp1-Luc) harboring a putative Pax5 binding site in the region from nt −768 to nt +240 (Figure 4A). In the Blimp1 luciferase reporter assay, Blimp1 promoter activity was inhibited (60.4–71.1% reduction, *p <* 0.01) in a dose-dependent manner by forced expression of Pax5 in RANKL-induced RAW264.7 cells (Figure 4B). The expression of antiosteoclastogenic factors, such as Bcl6, MafB, and IRF8, has been reported to be inhibited by Blimp1 expression during osteoclastogenesis [1,5]. Thus, we next examined whether the expression of antiosteoclastogenic factors is induced by Pax5 expression during osteoclastogenesis. Consistent with these results shown in Figure 4B, the expression of antiosteoclastogenic factors was significantly increased (Bcl6: 4.8-fold (*p <* 0.01), MafB: 1.8-fold (*p <* 0.01), and IRF8: 2.1-fold (*p <* 0.05) on day 4) by forced expression of Pax5 during RANKL-induced osteoclastogenesis (Figure 4C). These results indicate that Pax5 is crucial for the induction of antiosteoclastogenic factor expression through downregulation of Blimp1 in RANKL-induced osteoclastogenesis.

### 2.4. Osteoclastogenesis Is Reduced by Pax5 Transgene Expression in OC Lineage Cells

**Figure 5:**
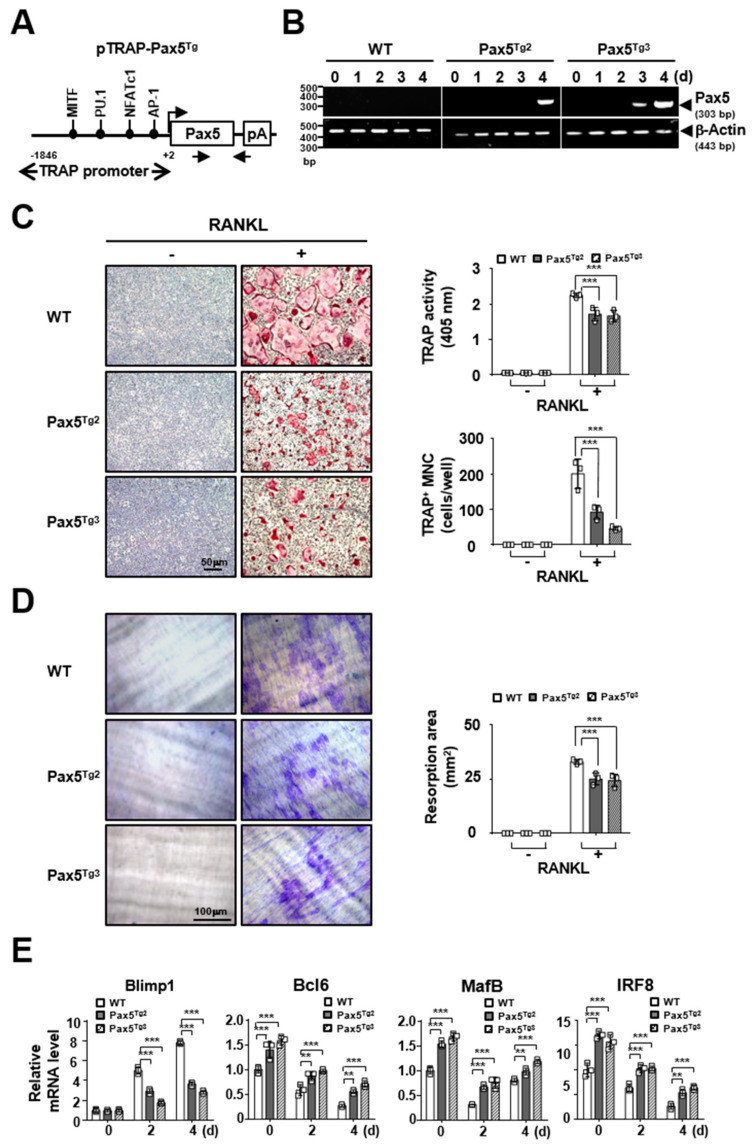
Pax5 transgene expression in OC lineage cells derived from Pax5 transgenic (Pax5^Tg^) mice inhibits osteoclastogenesis. (**A**) Schematic diagram of the Pax5 transgene expression vector. The transgenic vector (pTRAP-Pax5^Tg^) containing an OC lineage-specific *TRAP* promoter (−1846/+2), the murine Pax5 transgene, and an SV40 poly (A) signal sequence (pA) is shown. Nucleotide positions are numbered according to the transcription start site, and the putative binding sites of the transcription factors are marked. The arrows indicate the positions of the PCR primers used for genotyping. MITF, microphthalmia-associated transcription factor; AP-1, activation protein 1. (**B**) Expression of the Pax5 transgene in OCs derived from Pax5^Tg^ mice. Two Pax5Tg mouse lines (Pax5Tg2 and Pax5Tg3) were generated. BMMs derived from Pax5Tg2 and Pax5Tg3 mice were differentiated into OCs by treatment with M-CSF (50 ng/mL) and RANKL (100 ng/mL) for 4 days. Pax5 transgene expression in OCs was analyzed by semiquantitative RT-PCR. Wild-type (WT) mice were used as littermate control mice. β-Actin was used as the expression control. (**C**) Inhibitory effect of the Pax5 transgene on OC differentiation. BMMs derived from Pax5Tg2 and Pax5Tg3 mice were differentiated into OCs by treatment with M-CSF (50 ng/mL) and RANKL (100 ng/mL) for 4 days. The mature OCs were photographed after TRAP staining (left panel and original magnification, 50× (scale bar, 50 μm)). For the TRAP solution assay, the absorbance of OC samples was measured at 405 nm (top right panel). TRAP^+^ MNCs with more than three nuclei were counted (bottom right panel). Wild-type mice were used as littermate control mice. (**D**) Inhibitory effect of Pax5 transgene expression on resorption pit formation. Resorption pits on bone slices were visualized (left panel) by 1% toluidine blue staining (original magnification, 50× (scale bar, 100 μm)). The summary data from the resorption pit assays are shown in the right panel. (**E**) Real-time PCR analysis of Blimp1 and antiosteoclastogenic factors by Pax5 transgene expression. Total RNA was isolated in triplicate from OCs derived from Pax5Tg2 and Pax5Tg3 mice and analyzed by real-time PCR. The mRNA levels were normalized to those of β-actin. All points and error bars represent the mean ± SD of triplicate real-time PCRs. ** *p* < 0.01, *** *p* < 0.001.

To identify the effects of Pax5 transgene expression by OC lineage-specific control on osteoclastogenesis, we generated two Pax5Tg mouse lines (Pax5Tg2 and Pax5Tg3) through microinjection of a transgenic vector containing a human Pax5 transgene expression cassette under the control of the TRAP promoter (Figure 5A), which is expressed mainly in OC-specific lineage cells [16]. The levels of Pax5 transgene expression in OCs derived from BMMs of Pax5Tg2 and Pax5Tg3 mice were analyzed by semiquantitative RT-PCR (Figure 5B). We next analyzed the effects of Pax5 transgene expression on RANKL-induced osteoclastogenesis. BMMs derived from Pax5Tg mice were differentiated into OCs by RANKL stimulation. In the TRAP solution assay, TRAP activity was reduced (Pax5^Tg2^: 23.6% reduction and Pax5^Tg3^: 25.8% reduction on day 4, *p <* 0.001) in OCs derived from Pax5Tg mice compared to those derived from wild-type mice (Figure 5C, top right panel). We also observed that the size and number of TRAP^+^ MNCs were decreased (Pax5^Tg2^: 54.2% reduction and Pax5^Tg3^: 76.9% reduction on day 4, *p <* 0.001) in OCs derived from Pax5Tg mice (Figure 5C, left panel and bottom right panel). Consistent with these results, the resorption area of OCs derived from Pax5Tg mice was reduced (Pax5^Tg2^: 24.0% and Pax5^Tg3^: 26.2% reduction on day 4, *p <* 0.001) (Figure 5D). Lastly, we analyzed the levels of Blimp1 and antiosteoclastogenic factors in OCs derived from Pax5Tg mice using real-time PCR (Figure 5E). Similar to the results shown in Figure 2C and Figure 4C, the mRNA level of Blimp1 was significantly decreased in OCs derived from Pax5Tg mice compared to those derived from wild-type mice, while the levels of antiosteoclastogenic factors, such as Bcl6, MafB, and IRF8, were increased in OCs derived from Pax5Tg mice (Figure 5E). Taken together, these results indicate that Pax5 transgene expression via OC lineage-specific control is negatively involved in the regulation of RANKL-induced osteoclastogenesis.

### 2.5. Mice Expressing the Pax5 Transgene Show an Osteopetrotic-Like Bone Phenotype Caused by Reduced OC Formation

**Figure 6:**
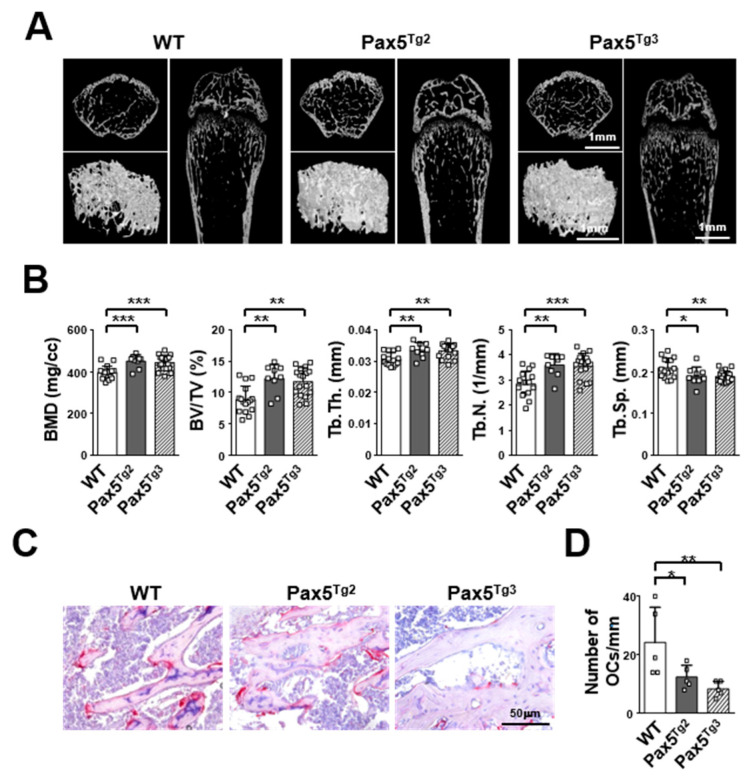
Mice expressing the Pax5 transgene exhibit an osteopetrotic-like bone phenotype. (**A**) Three-dimensional microstructural analysis of the distal femurs of Pax5 transgenic (Pax5^Tg^) mice. Femurs of Pax5^Tg^ mice (*n* = 10/group, 6 week old males) were fixed with 10% formaldehyde and analyzed by microcomputed tomography (micro-CT). Micro-CT images of the femurs are shown (scale bar, 1 mm). Wild-type (WT) mice were used as littermate control mice. (**B**) Three-dimensional trabecular structural parameters in distal femurs from Pax5^Tg^ mice. The following parameters were analyzed: bone mineral density (BMD), trabecular bone volume fraction (BV/TV), trabecular thickness (Tb.Th), trabecular number (Tb.N), and trabecular spacing (Tb.Sp). (**C**) The number of osteoclasts (OCs) in the distal femurs of Pax5^Tg^ mice was decreased. OCs in paraffin-embedded excised femur tissues were visualized by tartrate-resistant acid phosphatase (TRAP) staining, and the sections were then counterstained with hematoxylin (original magnification, 200× (scale bar, 50 μm)). (**D**) The number of TRAP^+^ OCs in the distal femurs of Pax5^Tg^ mice was decreased. The number of OCs in the femur was determined. * *p* < 0.05, ** *p* < 0.01, *** *p* < 0.001.

To examine the physiological role of Pax5 transgene expression in vivo, we next analyzed the bone phenotype of Pax5^Tg^ mice by microcomputed tomography (micro-CT) and bone histomorphometry. Three-dimensional images and bone parameters of the femoral trabecular and cortical bone in Pax5^Tg^ mice were measured and quantified using micro-CT (Figure 6A). The bone mineral density (BMD) of the trabecular bone in the femur of Pax5^Tg^ mice was significantly increased (Pax5^Tg2^: 13.6% higher (*p <* 0.001) and Pax5^Tg3^: 13.0% higher (*p <* 0.001)) compared to that in the femur of wild-type mice (Figure 6B, first panel). Consistent with these results, the trabecular bone volume fraction (BV/TV) was 32.5–38.1% higher (*p <* 0.01) in Pax5^Tg^ mice than in wild-type mice (Figure 6B, second panel). In addition, the trabecular thickness (Tb.Th) and trabecular number (Tb.N) were 8.4–9.0% higher (*p <* 0.01 or *p <* 0.01) and 25.5–26.7% higher (*p <* 0.01 or *p <* 0.001), respectively, in Pax5^Tg^ mice than in wild-type mice. However, the trabecular spacing (Tb.Sp) was 9.3–10.4% lower (*p <* 0.05 or *p <* 0.01) in Pax5^Tg^ mice than in wild-type control mice (Figure 6B, third through fifth panels). Next, we compared the number of TRAP^+^ OCs in the bone sections of the trabecular region of femurs obtained from Pax5^Tg^ and wild-type mice using TRAP staining and hematoxylin counterstaining. Consistent with the results shown in Figure 6A,B, the number of TRAP^+^ OCs was significantly decreased (Pax5^Tg2^: 48.8% reduction and Pax5^Tg3^: 65.3% reduction, *p <* 0.05 or *p <* 0.01) in Pax5^Tg^ mice compared to wild-type mice (Figure 6C,D). Hence, these results indicate that transgenic mice expressing the Pax5 transgene under the control of the TRAP promoter show an osteopetrotic-like bone phenotype.

## 3. Discussion

Pax5 is a well-known master transcriptional regulator of B-cell lineage specification and commitment [14,17]. Pax5 has also been shown to be a potential regulator of myeloid cell fate [14,17]. Forced expression of Pax5 in hematopoietic stem cells is sufficient to restrict T-cell lineage development in the thymus but not inhibit the development of myeloid lineage cells in bone marrow [18]. Indeed, Pax5^−/−^ splenocytes exhibit the characteristics of uncommitted multipotent stem cells, which can differentiate into multiple lineages of myeloid cells, such as macrophages, dendritic cells, and granulocytes [14,19]. Interestingly, Pax5 may also control OC cell fate and lineage commitment. The number and activity of OCs are increased in Pax5^−/−^ mice [14,15]. Furthermore, OCs can be differentiated from highly enriched OC precursors with increased cell surface expression of the M-CSF receptor (c-Fms) derived from Pax5^−/−^ splenocytes [14,15]. These findings raise the question of how Pax5 regulates cell fate determination and cellular potency related to OC lineage commitment in osteoclastogenesis. Thus, this study focused particularly on determining the mechanism via which OC lineage-specific Pax5 expression controls OC differentiation.

Pax5 contains a conserved DNA-binding PD, an octapeptide domain (OP), a partial homeodomain (HD), a transactivation domain (TA), and an inhibitory domain (ID) [7]. The PD is crucial for DNA binding to regulate the expression of downstream target genes [20,21]. This domain consists of an N- and a C-terminal subdomain, each of which binds independently to a distinct half-site in the recognition sequence [7,21]. Via the bipartite DNA binding of its PD subdomains, Pax5 binds to relatively degenerate DNA consensus sequences [22,23]. Thus, the binding of Pax5 to specific recognition sequences is potentially affected by interactions with other regulators that function as either transcriptional activators or repressors [14,21,24]. In our current study, we found that Pax5 acts as a negative regulator of osteoclastogenesis. Osteoclastogenesis was significantly inhibited by OC lineage-specific Pax5 expression (Figure 2 and Figure 5). Interestingly, we also showed that the inhibitory effect of Pax5 on osteoclastogenesis was partially abolished by deletion of its PD, while the inhibitory effect of Pax5 on osteoclastogenesis was not affected by deletion of the OP, HD, TA, or ID (Figure 3). Thus, according to our results, we postulate that the DNA-binding ability of Pax5 via the PD domain to its target consensus sequences is not absolutely required for its negative regulation of osteoclastogenesis. As DNA binding ambiguity in the PD subdomains of Pax5 has been revealed by previous structural studies [20,21,22,23], we cannot completely exclude the possibility that one or more other additional OC lineage-specific regulators that can interact with Pax5 are also involved in the negative regulation of osteoclastogenesis by Pax5. Pax5 has been reported to physically interact with several transcriptional regulators, such as groucho-related gene 4, Ets-1, PU.1, and runt-related transcription factor 1 [21,25,26,27]. Intriguingly, among these regulators, the functional roles of PU.1 as a transcriptional activator and RUNX1 as a transcriptional repressor in osteoclastogenesis have been identified [28,29]. Hence, further exploration of the transcriptional regulatory pathway linked to the negative regulation of osteoclastogenesis by Pax5 would be interesting.

Blimp1, a zinc finger motif-containing transcriptional repressor encoded by the *Prdm1* gene, is a key regulator of the terminal differentiation of plasma cells [17]. Blimp1 inhibits the expression of Pax5, which is crucial for B-cell lineage commitment and early B-cell development; in contrast, Pax5 represses Blimp1 expression through direct binding to exon 1 of the *Blimp1* gene, thereby inhibiting the terminal differentiation of plasma cells [10,11,12]. Thus, Blimp1 and Pax5 are reciprocal and antagonistic regulators of B-cell development and plasma-cell differentiation [11]. Interestingly, we observed that Pax5 expression was time-dependently downregulated by RANKL stimulation during osteoclastogenesis, whereas Blimp1 expression was enhanced, peaking on day 3 (Figure 1). Moreover, studies have demonstrated that Blimp1 induced by RANK–RANKL signaling represses the expression of antiosteoclastogenic factors, such as Bcl6, IRF8, and MafB, during osteoclastogenesis [1,30]. We, therefore, postulate that Pax5 negatively regulates Blimp1 expression during osteoclastogenesis. Indeed, we observed that both the expression level and the promoter activity of Blimp1 were clearly downregulated by Pax5 during RANKL-induced osteoclastogenesis, thereby repressing the expression of antiosteoclastogenic factors, such as Bcl6, IRF8, and MafB (Figure 2 and Figure 4). Consistent with previous studies on the crucial role of Pax5 in controlling cell fate decisions during B-cell development and plasma-cell differentiation, our findings suggest that the reciprocal and antagonistic regulation between Pax5 and Blimp1 ensures the specificity of the cell fate decision during RANKL-induced osteoclastogenesis. In addition, we interestingly observed increased trabecular thickness in Pax5^Tg^ mice compared to wild-type control mice (Figure 6B), indicating that the bone formation by osteoblasts is possibly enhanced in Pax5^Tg^ mice. Although we did not test the bone formation rate and osteoblast function in Pax5^Tg^ mice, we postulate that Pax5 transgene expression in OC lineage cells is linked to osteoblast differentiation and bone formation. Thus, further studies will be required to elucidate the role of Pax5 transgene expression in OC lineage cells affecting osteoblast differentiation and function.

In conclusion, we identified a negative role of Pax5 in osteoclastogenesis. Forced expression of Pax5 in OC lineage cells in vitro and in vivo inhibited RANKL-induced osteoclastogenesis. The inhibitory role of Pax5 in RANKL-induced osteoclastogenesis is mediated by downregulation of Blimp1 expression, resulting in the activation of antiosteoclastogenic factors, such as Bcl6, IRF8, and MafB. Thus, our data suggest that the regulation of the Pax5–Blimp1 axis is crucial for cell fate and lineage commitment decisions during RANKL-induced osteoclastogenesis.

## 4. Materials and Methods

### 4.1. Antibodies, Reagents, Cell Lines, and Mice

Specific antibodies were purchased from the following commercial sources: anti-Flag epitope and anti-β-actin were purchased from Sigma-Aldrich (St. Louis, MO, USA); anti-c-Fos and anti-NFATc1 were purchased from Santa Cruz Biotechnology (Dallas, TX, USA). Recombinant human soluble RANKL and human M-CSF were previously described [31,32]. RAW264.7 and PlatE cells were cultured in Dulbecco’s modified Eagle’s medium (Welgene, Daegu, Korea) supplemented with 10% fetal bovine serum (FBS) (Invitrogen, Carlsbad, CA, USA) and an antibiotic/antimycotic solution (Welgene) at 37 °C in a 5% CO_2_ atmosphere [31]. C57BL6/J mice were purchased from Daehan Biolink (Umsung, Korea) and maintained under pathogen-free conditions. Pax5^Tg^ mice expressing the human Pax5 transgene under the control of the TRAP promoter were generated by the Transgenic Core Facility of the Korea Advanced Institute of Science and Technology (Daejeon, Korea). The transgenic mice were backcrossed onto the C57BL6/J background for five generations. The genotype of Pax5^Tg^ mice was analyzed by PCR with the following primers: Pax5^Tg^ (sense), 5′–CCC TGC TGA CAT CGG GAG CAG T–3′; Pax5^Tg^ (antisense) 5′–GTC GTC ATC GTC TTT GTA GTC C–3′. All animal work was approved (approval no. CNU-00114, 1 January 2018) by the Animal Experiment Ethics Committee of Chungnam National University.

### 4.2. Plasmids

Retroviral expression plasmids for wild-type human Pax5 and its deletion mutants were constructed by PCR amplification and insertion into pMX-puro-Flag [32]. For construction of the Blimp1-Luc reporter vector, the region of the Blimp1 promoter from nt −768 to nt +240 was amplified by PCR from murine genomic DNA and subcloned into the pGL3-Basic vector (Promega, Madison, WI, USA). The plasmid pcDNA3.1HisLacZ was described previously [33]. The transgenic vector (pTRAP-Pax5^Tg^) containing an OC-specific TRAP promoter fragment from nt −1846 to nt +2, the human Pax5 transgene, and an SV40 poly(A) signal sequence was constructed by PCR amplification.

### 4.3. Osteoclastogenesis and OC Analysis

Bone marrow-derived OCs were prepared as described previously [31,32]. In brief, bone marrow cells collected from the tibia and femur of 8 week old C57BL6/J male mice were cultured in α-minimum essential medium (α-MEM) containing 10% FBS and M-CSF (5 ng/mL) for overnight. The cultured nonadherent bone marrow cells (1.5 × 10^5^ cells/well) in 96-well plates were differentiated into OCs in the presence of RANKL (100 ng/mL) and M-CSF (50 ng/mL) for 4 days. For retroviral transgene transduction and osteoclastogenesis, murine BMMs were prepared from 8 week old C57BL6/J male mice as described previously [34]. In brief, bone marrow cells isolated from mice were cultured in α-MEM containing 10% FBS and M-CSF (150 ng/mL) for 2 days. The cultured BMMs were infected by incubation with supernatants containing Pax5-expressing retroviruses along with polybrene (8 μg/mL) for 6 h and were then selected by incubation with puromycin (2 μg/mL) and M-CSF (150 ng/mL) for 2 days. The puromycin-resistant BMMs (1.5 × 10^4^ cells/well) in 96-well plates were differentiated into OCs in the presence of M-CSF (50 ng/mL) and RANKL (100 ng/mL) for 4 days. OCs were analyzed by TRAP solution and staining assays as previously described [34]. TRAP-positive multinucleated OCs (TRAP^+^ MNCs) with more than three nuclei were counted. For analysis of bone-resorbing activity, puromycin-resistant BMMs (1.5 × 10^4^ cells/well) were cultured in 96-well plates with M-CSF (50 ng/mL) and RANKL (100 ng/mL) on bone slices for 4 days. The bone slices were stained with 1% toluidine blue for 2 min and visualized under a microscope. The resorbing activity of OCs was evaluated by measuring the area of the resorption pit. To analyze OC marker gene expression, quantitative RT-PCR was performed in triplicate as previously described [34,35]. β-Actin expression was used as an internal control. The specific primers used were as follows: Pax5 (sense), 5′–AGA GAA AAA TTA CCC GAC TCC TC–3′; Pax5 (antisense), 5′–CAT CCC TCT TGC GTT TGT TGG TG–3′; TRAP (sense), 5′–AAA TCA CTC TTC AAG ACC AG–3′; TRAP (antisense), 5′–TTA TTG AAC AGC AGT GAC AG–3′; NFATc1 (sense), 5′–CCC AGT ATA CCA GCT CTG CCA TTG–3′; NFATc1 (antisense), 5′–GGA GCC TTC TCC ACG AAA ATG ACT–3′; DC–STAMP (sense), 5′–TCC TCC ATG AAC AAA CAG TTC CAA–3′; DC–STAMP (antisense), 5′–AGA CGT GGT TTA GGA ATG CAG CTC–3′; OSCAR (sense), 5′–TCT GCC CCC TAT GTG CTA TCA–3′; OSCAR (antisense), 5′–AGG AGC CAG AAC CTT CGA AAC–3′; cathepsin K (sense), 5′–ACG GAG GCA TTG ACT CTG AAG ATG–3′; cathepsin K (antisense), 5′–GTT GTT CTT ATT CCG AGC CAA GAG–3′; Blimp1 (sense), 5′–TTC TTG TGT GGT ATT GTC GGG ACT T–3′; Blimp1 (antisense), 5′–TTG GGG ACA CTC TTT GGG TAG AGT T–3′; Bcl6 (sense), 5′–AGA CGC ACA GTG ACA AAC CAT ACA A–3′; Bcl6 (antisense), 5′–GCT CCA CAA ATG TTA CAG CGA TAG G–3′; MafB (sense), 5′–TCA CAG AAA GAA CTC AGG A–3′; MafB (antisense), 5′–AAC GGT AGT GTG GAG GAC–3′; IRF8 (sense), 5′–AAG GTC ACC GTG GTC CTT AG–3′; IRF8 (antisense), 5′–GGA AAG CCT TAC CTG CTG AC–3′; β-actin (sense), 5′–ATG AAG ATC CTG ACC GAG CG–3′; and β-actin (antisense), 3′– TAC TTG CGC TGA GGA GGA GC–5′.

### 4.4. Luciferase Reporter Assay

Luciferase assays were performed as previously described [33,36]. In brief, RAW264.7 cells were seeded at 1 × 10^5^ cells/well into 24-well plates and transfected in triplicate with 0.1 μg of Blimp1-Luc, 0.2–1 μg of Flag-Pax5, and 0.13 μg of pcDNA3.1HisLacZ using TurboFect transfection reagent (Fermentas, Hanover, MD, USA). The transfected cells were further cultured with RANKL (100 ng/mL) for 2 days. Luciferase activity was measured using a luciferase assay system from Promega (Madison, WI, USA) and normalized to β-galactosidase activity.

### 4.5. Micro-CT and Histological Analysis

Femurs from Pax5^Tg^ mice (6 week old males, *n* = 10 per group) were fixed with 10% formaldehyde, and trabecular morphometry of distal femurs was performed using micro-CT (SkyScan 1076, Bruker micro-CT, Kontich, Belgium) at 6.775 μm image pixel resolution with 50 kV, 200 μA, and a 0.5 mm aluminum filter. Image reconstruction was performed with the software interface (Nrecon 1.6.1.5), which was provided by the manufacturer of the scanner, with a smoothing of 1, ring artefact of 8, and beam hardening of 30%. Reconstructed images were realigned in Dataviewer software 1.5.2 (Bruker micro-CT, Kontich, Belgium). Bone phenotypes were analyzed by measuring the BMD, BV/TV, Tb.Th, Tb.N, and Tb.Sp in the region of interest using 50 slices approximately 0.4 mm away from the growth plate of the distal femur, as previously described [37]. For histological analysis, the fixed femurs were decalcified in 15% ethylenediaminetetraacetic acid solution at 4 °C for 21 days with mild agitation prior to embedding in paraffin, as previously described [32]. Paraffin sections (7 μm) were sliced and subjected to TRAP staining and hematoxylin counterstaining. The TRAP^+^ OCs in the femur were counted by visualization under a microscope.

### 4.6. Statistical Analysis

Data are expressed as the mean ± SD of values from at least three independent experiments. Statistical analyses were performed using a two-tailed Student’s *t*-test to analyze differences between two groups or one-way and two-way ANOVA, if there was one or more than two conditions, respectively, followed by Dunnett’s multiple comparisons test to evaluate differences among at least three groups. A *p*-value < 0.05 (*) was considered statistically significant.

## Data Availability

The data presented in this study are available within this article.

## Abbreviations

Pax5	Paired box protein 5
Blimp1	B lymphocyte-induced maturation protein 1
RANKL	Receptor activator of nuclear factor kappa B ligand
M-CSF	Macrophage-colony stimulating factor
BMM	Bone marrow-derived macrophage
NFATc1	Nuclear factor of activated T cells c1
TRAP	Tartrate-resistant acid phosphatase
DC-STAMP	Dendritic cell-specific transmembrane protein
OSCAR	Osteoclast-associated receptor
Bcl6	B-cell lymphoma 6
IRF8	Interferon regulatory factor 8
MafB	v-Maf musculoaponeurotic fibrosarcoma oncogene family homolog B
